# Neurophysiological hallmarks of Huntington’s disease progression: an EEG and fMRI connectivity study

**DOI:** 10.3389/fnagi.2023.1270226

**Published:** 2023-12-15

**Authors:** Natalya V. Ponomareva, Sergey A. Klyushnikov, Natalia Abramycheva, Rodion N. Konovalov, Marina Krotenkova, Ekaterina Kolesnikova, Daria Malina, Gusel Urazgildeeva, Elena Kanavets, Andrey Mitrofanov, Vitaly Fokin, Evgeny Rogaev, Sergey N. Illarioshkin

**Affiliations:** ^1^Research Center of Neurology, Moscow, Russia; ^2^Center for Genetics and Life Science, Sirius University of Science and Technology, Sochi, Russia; ^3^Research Center of Mental Health, Moscow, Russia; ^4^Department of Psychiatry, Umass Chan Medical School, Shrewsbury, MA, United States

**Keywords:** EEG, functional MRI, Huntington’s disease, preclinical stage, HTT gene, CAG repeats, cognitive functions

## Abstract

Electroencephalography (EEG) and functional magnetic resonance imaging (fMRI) can provide corroborative data on neurophysiological alterations in Huntington’s disease (HD). However, the alterations in EEG and fMRI resting-state functional connectivity (rsFC), as well as their interrelations, at different stages of HD remain insufficiently investigated. This study aimed to identify neurophysiological alterations in individuals with preclinical HD (preHD) and early manifest HD (EMHD) by analyzing EEG and fMRI rsFC and examining their interrelationships. We found significant differences in EEG power between preHD individuals and healthy controls (HC), with a decrease in power in a specific frequency range at the theta-alpha border and slow alpha activity. In EMHD patients, in addition to the decrease in power in the 7–9 Hz range, a reduction in power within the classic alpha band compared to HC was observed. The fMRI analysis revealed disrupted functional connectivity in various brain networks, particularly within frontal lobe, putamen-cortical, and cortico-cerebellar networks, in individuals with the HD mutation compared to HC. The analysis of the relationship between EEG and fMRI rsFC revealed an association between decreased alpha power, observed in individuals with EMHD, and increased connectivity in large-scale brain networks. These networks include putamen-cortical, DMN-related and cortico-hippocampal circuits. Overall, the findings suggest that EEG and fMRI provide valuable information for monitoring pathological processes during the development of HD. A decrease in inhibitory control within the putamen-cortical, DMN-related and cortico-hippocampal circuits, accompanied by a reduction in alpha and theta-alpha border oscillatory activity, could potentially contribute to cognitive decline in HD.

## Introduction

Huntington’s disease (HD) is a progressive neurodegenerative disorder characterized by gradual development of invalidating motor, cognitive, and psychiatric symptoms. Frontal-subcortical dementia is a common manifestation of HD and typically emerges in the later stages of the condition (Martinez-Gutierrez et al., 2022).

HD is an autosomal dominant disease caused by an abnormal expansion of CAG repeats in the huntingtin gene (*HTT*) located on chromosome 4p16.3 (The Huntington’s Disease Collaborative, Research Group, 1993; Warby et al., 2009). The length of the CAG repeats in the *HTT* gene has been established as a major determinant of disease onset and progression, with longer repeat expansions generally leading to an earlier age of symptom onset (Stine et al., 1993; Illarioshkin et al., 1994; Penney et al., 1997). The toxicity of mutant huntingtin (mHTT) affects various cellular processes, including mitochondrial function, the expression of neurotrophic factors and G protein-coupled receptors, vesicular trafficking in synaptic neurotransmission, and synaptic plasticity (Cisbani and Cicchetti, 2012).

HD is characterized by a long preclinical phase, during which affected individuals carry the gene mutation but do not yet exhibit overt clinical symptoms. The transition from the preclinical phase to manifest motor symptoms is marked by significant neurodegeneration and progressive functional alterations.

There is an increasing need to identify reliable biomarkers that can facilitate early detection and the monitoring of disease progression especially in preclinical stage. The emergence of therapies targeting the reduction in huntingtin synthesis makes the identification of biomarkers in HD particularly important.

Electroencephalography (EEG) is a noninvasive technique that detects changes in brain oscillatory activity, indicative of alterations caused by synaptic dysfunction and progressive neurodegeneration in HD. In patients with manifest HD, previous studies have shown alterations in EEG power, including increased delta and decreased alpha power (Streletz et al., 1990; Bylsma et al., 1994; Painold et al., 2010, 2011; Hunter et al., 2010; Nguyen et al., 2010 for review; Leuchter et al., 2017; Piano et al., 2017; Odish et al., 2018; Delussi et al., 2020; Davis et al., 2023). In premanifest HD (PreHD) and early manifest HD (EMHD), a decrease in power in the low alpha band and at the theta-alpha border has been reported (Painold et al., 2010, 2011, 2017; Ponomareva et al., 2014; Lazar et al., 2015; Leuchter et al., 2017; Hawellek et al., 2022).

This particular EEG frequency range has been suggested as a valuable tool for monitoring changes in brain function in Huntington’s Disease (HD). It can be especially useful in assessing the effectiveness of treatments aimed at reducing levels of the huntingtin protein, which are currently undergoing clinical development. Treatment with the huntingtin-lowering oligonucleotide tominersen has demonstrated a partial restoration of activity within this frequency range (Hawellek et al., 2022). Collectively, these findings show the informative potential of this understudied frequency range in assessing brain activity in individuals with PreHD and EMHD.

Previous studies have described the association between the number of CAG repeats in the *HTT* gene and EEG alterations in preclinical and early manifest HD (Bellotti et al., 2004; Hunter et al., 2010; Ponomareva et al., 2014). EEG alterations have been found to be associated with worse cognitive performance in both preclinical and symptomatic individuals with HD (Painold et al., 2011; Ponomareva et al., 2014; Piano et al., 2017; Odish et al., 2018; Delussi et al., 2020).

The investigation of the influence of impairment in subcortical networks, including those of the striatum, is of interest not only in HD but also in other neurodegenerative diseases. It has been shown that in individuals with mild cognitive impairment (MCI), the gray matter density of the basal ganglia and thalamus, estimated using voxel based morphometry, was associated with the EEG alpha2/alpha3 ratio (Moretti et al., 2012).

Functional magnetic resonance imaging (fMRI) is a powerful technique that utilizes the blood oxygenation level dependent (BOLD) signal to measure changes in blood oxygenation levels in brain vessels, allowing the inference of regional neural activity and functional connectivity patterns.

fMRI resting-state functional connectivity (rsFC) has been extensively used to investigate alterations in functional connectivity in preclinical and symptomatic HD (Dumas et al., 2013; Quarantelli et al., 2013; Harrington et al., 2015; Poudel et al., 2015; Wolf et al., 2015; Sarappa et al., 2017; Espinoza et al., 2018; Kronenburger et al., 2019; Nair et al., 2022). These studies have consistently demonstrated disrupted connectivity within the cortico-striatal and cortico-cortical networks. These connectivity changes are thought to reflect the underlying brain dysfunction neurodegenerative processes and contribute to the manifestation of clinical symptoms in HD.

The results of fMRI rsFC studies in HD may vary due to differences in participants groups and research methods. Many studies have demonstrated reduced connectivity in patients with prodromal and manifest HD, particularly in striato-cortical circuits and motor regions (Dumas et al., 2013; Werner et al., 2014; Pini et al., 2020). However, hyperconnectivity has also been reported, suggesting the presence of compensatory mechanisms (Wolf et al., 2012; Kronenburger et al., 2019; Pini et al., 2020 for review).

Although EEG and fMRI capture different aspects of brain activity, their combination can provide a better understanding of the neural alterations in HD. This integrated approach holds promise for identifying specific biomarkers that correlate with disease progression and could be used to assess the efficacy of potential disease-modifying therapies. The relationship between changes in EEG power and alterations in fMRI connectivity within neural networks in HD remains insufficiently investigated. The study of this interrelation will enable a better understanding of the neural networks involved in the alteration of EEG characteristics in preclinical and early-manifest HD.

*This study aimed to identify neurophysiological alterations in individuals with preclinical HD (preHD) and early manifest HD (EMHD) by analyzing EEG and fMRI resting state functional connectivity (rsFC) and examining their interrelationships*.

We are focusing on the interrelation between alpha relative power and fMRI rsFC, as the alpha rhythm has been previously identified as the most affected in HD.

In our study, we investigated the neurophysiological alterations in preclinical and early-manifested HD by using both rsFC fMRI and EEG characteristics on the same subjects.

In cases where subjects are in a resting state with relatively stable conditions, it is considered acceptable to conduct separate EEG and fMRI recordings in distinct experimental sessions (Scrivener, 2021).

## Materials and methods

### Participants

All subjects were predominantly Russian from Moscow and the Moscow region. The enrolled cohort included 56 *HTT* mutation carriers (27 men, 29 women), a mean age 32.5 ± 1.1 years with a CAG repeat length of 43.9 ± 0.5 and 41 age-matched healthy subjects (15 men, 26 women), mean age of 32.8 ± 1.7 years. The group *HTT* mutations carriers comprised 45 individuals with PreHD (19 men, 26 women), a mean age of 31.4 ± 1.1 years with a CAG repeat length of 43.2 ± 0.5; and 11 individuals with EMHD (8 men and 3 women), a mean age of 36.9 ± 2.9 years with a CAG repeat length of 46.8 ± 1.5. The disease burden score (DBS) giving a measure of toxic load was calculated for each pre-HD participant using the formula: (CAG repeat length — 35.5) × age (Tabrizi et al., 2012).

“Preclinical” in this article denotes the absence of unequivocal motor signs as assessed with the Unified Huntington’s Disease Rating Scale (UHDRS) (Huntington Study Group, 1996), established during a subject’s most recent visit to the outpatient clinic of the Research Center of Neurology (Moscow). That visit took place no longer than one week before the EEG and neuropsychological examinations. All participants earlier underwent predictive DNA testing. Mutation carriers were defined as persons having more than 36 CAG repeats in one allele of the *HTT* gene. PreHD was defined as individuals with *HTT* CAG repeat expansions >36 and a the diagnostic confidence level (DCL) < 2 of the Total Motor Score (TMS) as part of the UHDRS, whereas EMHD was defined as individuals with an *HTT* CAG repeat expansion >36 and a DCL = 4. All examined EMHD patients had >40 *HTT* CAG repeat.

All patients with EMHD were at stage 2 of the Huntington’s Disease Integrated Staging System (HD-ISS) (Tabrizi et al., 2022). Among the 45 PreHD individuals who underwent EEG examinations, 40 subjects were at stage ≤1 of the HD-ISS. Five individuals with PreHD did not meet the HD-ISS criteria due to having a CAG expansion in the range of 37–39 (three subjects had 39, one had 38, and one had 37 CAG repeats in the HTT gene). Among the 19 PreHD individuals who underwent both fMRI and EEG examinations, 17 were at stage ≤1 of the HD-ISS. Two HD patient relatives with PreHD did not meet the HD-ISS criteria due to having 39 CAG repeats. Control analysis, excluding *HTT* mutation carriers with 37–39 CAG repeats, confirmed all the received results.

Exclusion criteria were neurological and psychiatric comorbidity, any medication, as well as the presence of clinical HD based on an unequivocal motor diagnosis on the UHDRS.

Healthy controls were individuals with no relatives having HD or other neurodegenerative diseases. Controls underwent neurological examination and cognitive screening, and were found to be free of dementia and other medical, psychiatric and neurological conditions, including cerebrovascular diseases, hypertension, epilepsy and endogenous disorders. Exclusion criteria were a history of neurological and psychiatric diseases and any kind of memory impairment. Pre-HD subjects and HC also underwent a neuropsychological battery that included the following tests: the Mini Mental State Examination (MMSE) (Folstein et al., 1975), the modified pictures memory test (van der Hiele et al., 2007), the Controlled Oral Word Association Test (FAS) (Benton and Hamsher, 1989), Luria memory words test (LMWT) (Luria, 1966),and the Spielberger state–trait anxiety inventory (STAI) (Spielberger, 1983), the Total Functional Capacity (TFC) from the UHDRS, Symbol Digit Modalities Test (SDMT) (Smith, 1982), Stroop Word Reading Test (Stroop, 1935).

### EEG recording and data acquisition

#### EEG procedure

The registration and evaluation of EEG was carried out in accordance with the IPEG guidelines (Versavel et al., 1995; Jobert et al., 2012). EEGs were recorded during resting state for a period of 3 min. The subjects were instructed to sit comfortably in a chair, and to close their eyes and relax during the recording, but to remain awake. The technician watched the subject’s vigilance state continuously by monitoring the EEG and observing the subject. He verbally alerted the subject any time there were signs of behavioral and/or EEG drowsiness or the subject opened the eyes. These periods were labeled and excluded from data analysis.

EEG was recorded on Nihon Kohden 4,217 G EEG using the time constant of 0.3 s. The high frequency cut-off was 40 Hz. The 16 Ag/AgCl electrodes were placed according to the international 10–20 system at O2, O1, P4, P3, C4, C3, F4, F3, Fp2, Fp1, T6, T5, T4, T3, F8, and F7 positions. Linked ears served as the reference. Electrode impedance did not exceed 10 kΩ. EEG was continuously recorded on paper, and the experimenter did the conventional visual inspection of the entire EEG records. During the recording, 180 s of EEG was simultaneously sampled at 256 Hz per channel and stored on a computer for further analysis offline. The EEG was reviewed visually for artifacts. Periods of artifact were eliminated from subsequent analysis. After the elimination of artifacts, segments of resting EEG of 120-s duration were selected for further analysis.

Frequencies below 2 Hz and above 35 Hz were eliminated by digital filtering. Thirty 4-s artifact-free epochs of resting EEG were processed by fast Fourier transform. The obtained spectra were averaged to obtain a mean power spectrum for each channel, which was divided into 4 bands. The relative power (% of total EEG power) of the delta (2.00–3.99 Hz), theta (4.00–7.99), alpha (8.00–12.99), beta1 (13.00–19.99), and beta2 (20.00–30.00) bands, as well as of every 1-Hz sub-band of the theta and alpha bands (4–13 Hz) were calculated. Log transformations of the relative power of the various bandwidths in each derivation were calculated in order to compensate for data skewedness, as recommended by John et al. (1980), using log [*x*/(1 − *x*)], where *x* is the fraction of total power for each 4-s sample. The log relative power values for each examined frequency range were obtained by averaging across electrodes. The details of the spectral analysis procedures have been previously described (Ponomareva et al., 2014, 2022).

#### Genetic analysis

DNA was isolated from peripheral blood lymphocytes using a Promega Wizard Genomic DNA Purification Kit, according to the manufacturer’s instructions. The *HTT* exon 1 CAG repeat length was determined by fragment analysis using the following primer pair: 5′-(FAM) GCGACCCTGGAAAAGCTGAT-3′ (forward) and 5’-GGTGGCGGCTGTTGCTGCTGC-3′(reverse). Polymerase chain reaction (PCR) with these primers does not amplify CCG repeat adjacent to the region of CAG repeat (Andrew et al., 1994). PCR products were analyzed on an ABI 3130 sequencer using LIZ-500 (ABI) as a standard. Analysis was performed using GeneMapper v4.0.Alleles with 38 or more repeats were considered expanded.

### fMRI imaging acquisition

In addition to other examinations, fMRI rsFC was examined in a subgroup of 29 *HTT* mutation carriers (14 men, 15 women), a mean age 35.2 ± 1.4 years with a CAG repeat length of 44.1 ± 0.7 and 25 age-matched healthy subjects (8 men, 17 women), a mean age 38.0 ± 1.9 years. The group *HTT* mutations carriers included 19 PreHD individuals (7 men, 12 women), a mean age 34.8 ± 1.4 years with CAG repeat length of 43.0 ± 0.6; and 10 individuals with EMHD (7 men and 3 women), a mean age of 36.0 ± 3.0 years with a CAG repeat length of 46.3 ± 1.5.

Structural images were acquired using a T1-weighted MPRAGE sequence: TR = 1,900 ms, TE = 2.47 ms; FOV = 256 × 256 mm2; flip angle = 10° slice thickness 1.0 mm; interslice distance 1 mm; number of slices = 176.

Functional scans were obtained at rest using T2_-weighted EPI sequence: TR = 1,500 ms, TE = 30 ms, flip angle 70°, slice thickness 2 mm, FOV = 190 mm, FoV phase 100.0%. The subjects were instructed to relax as much as possible, to lie quietly with their eyes closed (to exclude stimulation of the visual system) and not to think about anything in particular.

For analysis of rsFC signals in fMRI images we used CONN, which is a MATLAB-based open source toolbox (Functional Connectivity SPM Toolbox 2017, McGovern Institute for Brain Research, Massachusetts Institute of Technology) (Whitfield-Gabrieli and Nieto-Castanon, 2012).

CONN toolbox version 18b in conjunction with the SPM 12 software package (Wellcome Department of Cognitive Neurology, London, United Kingdom, www.fil.ion.ucl.ac.uk/spm) was used to perform all preprocessing steps. Functional images were slice-time corrected, realigned (motion corrected), and coregistered to their respective T1-weighted anatomical image. Images were then normalized to the Montreal Neurological Institute (MNI) standard space and spatially smoothed with an 8-mm Gaussian filter.

Denoising methods was then applied to minimize the impact of artifactual sources of signal variability. This included band-pass filtering (0.01–0.1 Hz), scrubbing (volumes showing displacement larger than the 97 × th percentile were censored), regressing out of the first 10 principal components (aCompCor) calculated within the maps of white matter (five components) and cerebrospinal fluid (five components) and regressing out of 24 head motion parameters, including linear and rotational indices, their temporal derivatives and their squared values.

The CONN-18.b toolbox was used to obtain a linear measure of functional connectivity based on bivariate correlation and bivariate regression coefficients between seed areas for ROI-to-ROI analysis (Whitfield-Gabrieli and Nieto-Castanon, 2012). ROIs of the whole brain were drawn from the template provided by CONN (conn/rois/atlas.nii). For the purpose of the analysis, BOLD signal time courses were converted to normally distributed scores with Fisher’s transformation, which allows for the use of second-level general linear Model analysis.

We performed a region of interest (ROI) analysis whereby ROIs were anatomically defined using the FSL Harvard-Oxford maximum probability cortical atlas, with bilateral regions divided into left and right hemispheres (164 ROIs).

### Statistical analysis

Differences in demographic scores between the groups (*HTT* mutation carriers vs. HC; PreHD vs. HC; EMHD vs. HC; PreHD vs. EMHD) were tested using analysis of variance (ANOVA) for continuous variables (age, education), and the Mann–Whitney U test for categorical variables (sex). EEG parameters from each group were tested for normal distribution by the Wilk–Shapiro test, and in no cases were the data skewed.

The significance of the differences between the log-transformed EEG parameters was estimated using repeated measures of ANOVA in the general linear model (GLM), with group (*HTT* mutation carriers vs. HC; PreHD vs. HC; EMHD vs. HC; PreHD vs. EMHD as a between-subjects factor, and bands as a within-subject factor; age and gender were included in the analysis as covariates. Separate estimations using repeated measure ANOVA were performed on the EEG, firstly for the traditional broad bands, and subsequently for the 1-Hz subbands of theta and alpha (4–13 Hz). Post-hoc comparisons for between-subject effects and within-subject effects were analyzed using the Duncan test, and the level of significance was set to *p* < 0.05.

During fMRI rsFC analysis, our primary focus was on differences between two groups, *HTT* mutation carriers and HC, using t-tests, considering the smaller sample size compared to the EEG analysis. As the groups of the *HTT* mutations and HC had nonsignificant differences in gender and age, we did not include these factors in the analysis.

We compared ROI-to-ROI rsFCs between the *HTT* mutation carriers vs. HC using two-tailed t-tests. The resulting statistical maps were set with p < 0.05 false discovery rate (FDR) corrected.

In addition, we used one-sample t-test to assess rsFCs in the HC group. A similar analysis was conducted using one-sample t-test to evaluate rsFCs in the group of *HTT* mutation carries. In all analysis, significance was determined using two-tailed t-tests, with a significance level set at p < 0.05 FDR corrected. The analysis specifically was focused on rsFCs within circuits where a significant difference between *HTT* mutation carriers and HC had already been found.

To investigate associations between CAG repeat numbers in *HTT* gene, DBS, EEG characteristics and psychometric measures, Pearson correlation analysis was used whenever the data followed a normal distribution and Spearman rank correlations were calculated in other cases. The results were adjusted for multiple comparisons by applying FDR corrections at *p* < 0.05 using the Benjamini and Hochberg (1995). We conducted a correlation analysis, including only the relative power of the standard EEG bands (delta, theta, alpha. beta1, beta2) and the 1-Hz ranges (specifically 7–8 Hz, 8–9 Hz and the difference between 7–8 and 4–5 Hz), as these frequencies have shown significant differences in PreHD, EMHD and HC. An uncorrected significance level of p < 0.05 was considered to indicate a tendency.

Regression analyses between fMRI ROI-ROI connectivity strength and EEG alpha relative power were conducted directly in the Conn Toolbox using generalized PsychoPhyisiological Interaction analysis. The results were assessed utilizing an analysis with an FDR correction at a significance level of *p* < 0.05. This correction took into account the total number of pairwise correlations run in our ROI-ROI analysis (164×163/2), and computes an FDR correction accordingly. We examined only the interrelation between alpha relative power and fMRI rsFC, as the alpha rhythm has been previously identified as the most affected in HD.

## Results

The demographic and cognitive characteristics of the subjects are shown in Table 1. The participant groups were not significantly different in age, sex or education when assessed using one-way analyses of variance and *χ*^2^ tests.

**Table 1:** Demographic and psychometric characteristics of participants.

			*HTT* mutation carriers					Healthy control
	PreHD group	*P* PreHC vs. HC	EMHD group	*p* EMHD vs. PreHD	*p* EMHD vs. HC	Combined PreHD & EMHD	*p* HC vs. PreHD&EMHD	
N	45		11			56		41
Age, years (SE)	31.4 (1.1)	0.48	36.9 (2.9)	0.04	0.25	32.5 (1.1)	0.9	32.8 (1.7)
Male/female	19/26	0.6	8/3^a^	0.07	0.03	27/29	0.3	15/26
Education, years (SE)	14.4 (0.2)	0.13	14.3 (0.4)	0.9	0.25	14.4 (0.2)	0.1	14.8 (0.2)
CAG repeats (SE)	43.26 (0.5)		46.8^b^ (1.5)	0.003		43.9 (0.5)		−
DBS (SE)	231.1 (12.7)		386.4^b^ (37.9)	<0.001		261.6 (15.0)		−
FAS, words (SE)	46.5 (2.0)	0.07	33.1^a,^^b^ (2.6)	0.004	<0.001	44.0^a^ (1.9)	0.006	51.5 (1.8)
LMWT (SE)	5.8 (0.3)	0.23	4.7^a,^^b^ (0.4)	0.06	0.006	5.6 (0.2)	0.06	6.2 (0.2)
LMWT total score (SE)	38.6^a^ (1.3)	<0.001	33.4 ^a,b^ (1.9)	0.03	<0.001	37.1^a^ (1.1)	<0.001	44.2 0.6
Pictures total score (SE)	35.4^a^ (0.6)	0.003	29.4^a^^,^^b^ (2.2)	<0.001	<0.001	34.5^a^ (0.6)	<0.001	38.0 (0.6)
MMSE (SE)	28.8 (0.6)	0.07	27.1^a^^,^^b^ (0.8)	0.10	<0.001	28.2^a^ (0.5)	0.008	29. (0.1)
STAI state score (SE)	38.8 (1.4)	0.09	35.6 (3.4)	0.33	0.97	38.2 (1.3)	0.16	35.5 (1.4)
STAI trait score (SE)	41.8 (1.7)	0.42	38.2 (4.1)	0.37	0.6	41.1 (1.6)	0.62	40.0 (1.4)
UHDRS-TMS (SE)	1.3 (0.5)	0.011	18.5^a^^,^^b^ (2.7)	<0.001	<0.001	8.4^a^ (2.4)		0
SDMT (SE)	45.6^a^ (3.1)	<0.001	27.6^a^^,^^b^ (2.9)	0.0005	<0.001	38.6^a^ (2.7)	<0.001	59.4 1.6
Stroop color naming (SE)	75.1 (3.7)	0.22	56.2^a^^,^^b^ (3.4)	0.001	<0.001	67.4^a^ (3.1)	0.003	80.3 (2.1)
Stroop word reading (SE)	86.5 (3.2)	0.14	70.3^a^^,^^b^ (4.8)	0.007	<0.001	80.3^a^ (3.0)	0.005	92.4 (2.3)
Stroop interference (SE)	42.8 (2.4)	0.63	34.9^a^^,^^b^ (2.3)	0.03	0.015	39.7 (1.8)	0.11	44.5 (2.5)
TFC (SE)	13.0 0.00		12.2^a^^,^^b^ (0.3)	0.001	<0.0001	12.7^a^ (0.1)	0.04	13.0 0.00

Data are presented as the means and standard errors (SE). MMSE, Mini-Mental State Examination; LMWT, Luria Memory Words Test; FAS, Controlled Oral Word Association Test; SST, STAI, Spielberger State–Trait Anxiety Inventory, SDMT, Symbol Digit Modalities Test; TFC – total functional capacity.

a significant differences *p* < 0.05 between healthy control and HTT mutation carriers.

b significant differences *p* < 0.05 between PreHD individuals and EMHD patients.

In the group of *HTT* mutation carriers, which included preHD individuals and EMHD patients, scores for semantic verbal fluency (FAS), LMWT and working memory scores, SDMT and Stroop Word Reading Test, TFC and MMSE were lower than those in the HC subjects. The STAI state and STAI trait scores were not significantly different among these groups. The PreHD individuals exhibited lower SDMT and LMWT total scores than the HC subjects, while the two groups had similar results in the other neuropsychological tests.

The patients with EMHD showed lower scores in all cognitive tests than the HC subjects and the PreHD individuals (Table 1).

### Comparison of EEG among a group of individuals carrying an *HTT* mutation, including those with PreHD and EMHD, and a control group of healthy individuals

**Figure 1:**
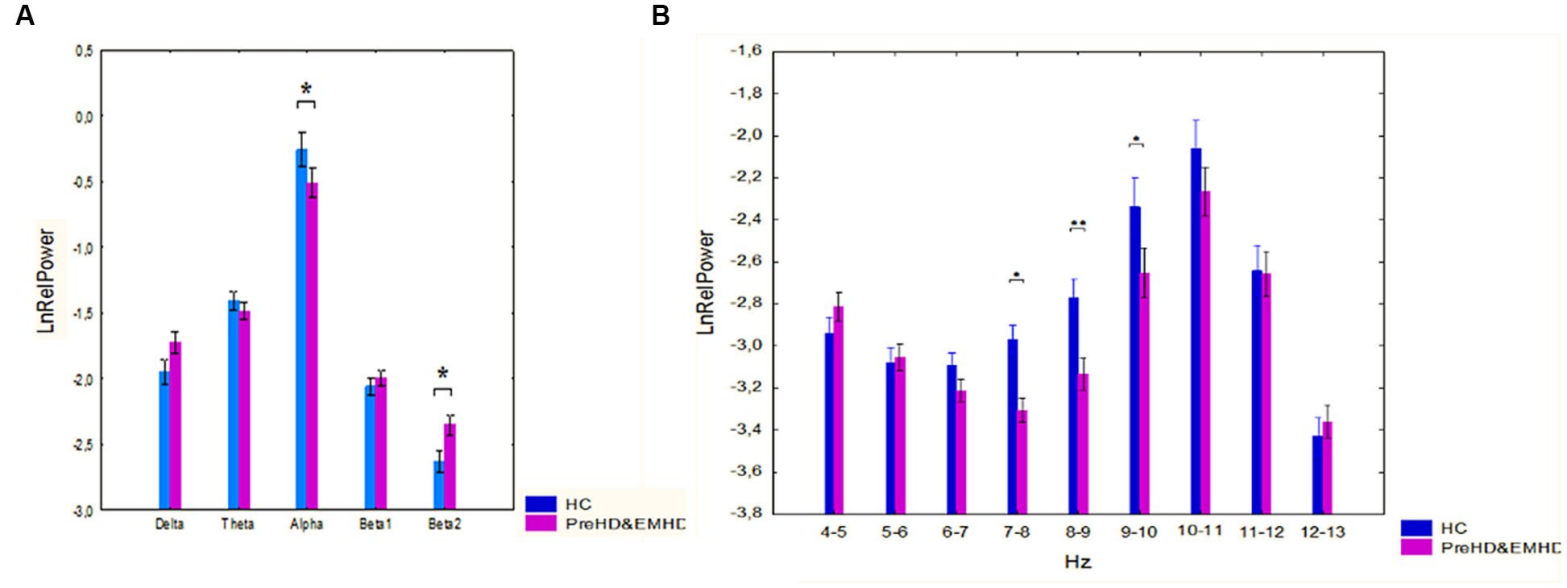
Log-transformed relative power of EEG bands (mean and standard error [SE]) in individuals with *HTT* mutations, including those with PreHD and EMHD, and the control group of healthy individuals (HC). **(A)** Standard broad frequency bands. **(B)** Every 1-Hz subband of theta and alpha bands. * *p* < 0.05, **p < 0.01: Significant differences in relative power of EEG frequency bands or subbands between the *HTT* mutations carriers and healthy individuals.

The ANOVA results showed a significant interaction between band and groups (F[4,352] = 2.79, *p* = 0.026). Post-hoc comparison showed that the alpha relative power was decreased (*p* = 0.038) and the beta2 relative power was increased (*p* = 0.018) in *HTT* mutation carriers as compared to HC individuals (Figure 1A).

ANOVA revealed a robust effect of group, *HTT* mutation carriers vs. HC individuals, on the relative power of the 1 Hz spectral frequency bands in the range 4–13 Hz (F(Alper et al., 2006; Zhang et al., 2023) = 11.56, *p* = 0.001). The ANOVA results also showed a Group x Band interaction (F[8, 704] = 2,07, *p*=,037). In the group of individuals carrying *HTT* mutations, the relative power of the 7–8 Hz, 8–9 Hz and 9–10 Hz frequency bands was reduced compared to that in the HC individuals (*p* = 0.016, *p* = 0.008 and *p* = 0.017 for the 7–8 Hz, 8–9 Hz and 9–10 Hz frequency bands respectively) (Figure 1B).

**Figure 2:**
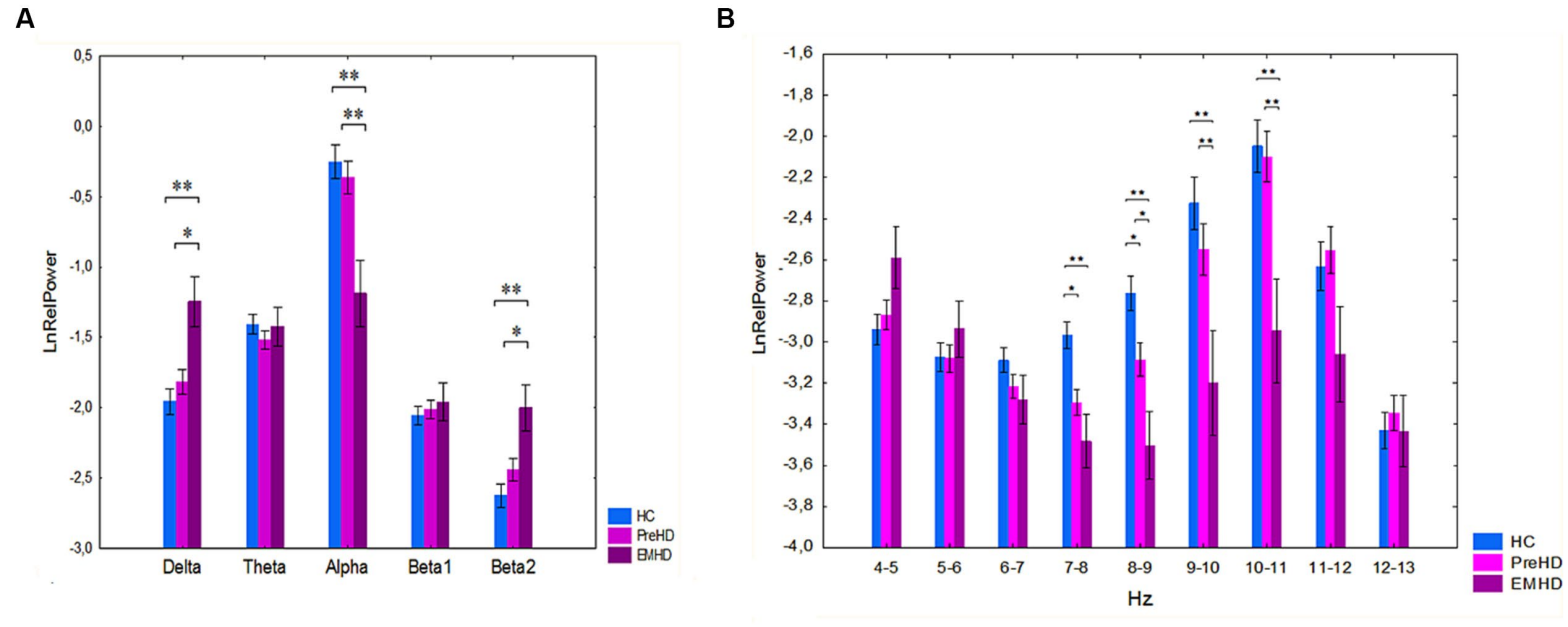
Log-transformed relative power of EEG bands (mean and standard error) in PreHD, EMHD and HC individuals. **(A)** Standard broad frequency bands. **(B)** Every 1-Hz subband of theta and alpha bands. *p < 0.05, **p < 0.01: Significant difference in log-transformed relative power of EEG frequency bands and subbands between the EMHD, PreHD and healthy individuals.

We performed a further analysis of the differences in EEG spectral power separately for the PreHD, EMHD, and HC groups. A significant interaction effect was observed between the group and band factors on the relative power of traditional broad EEG bands (F[8,364] = 5.0 p=,00001) (Figure 2A). *Post hoc* comparison indicated that there was no significant difference between the PreHD and HC groups. *Post hoc* comparison showed that in the EMHD group compared to the HC group, the delta relative power was increased (*p* = 0.001), the alpha relative power was reduced (*p* = 0.00002) and the beta2 relative power was increased (*p* = 0.0004). In the EMHD group delta power was higher (*p* = 0.03), alpha power was lower (*p* = 0.0002), and beta2 power was higher (*p* = 0.01) than in the PreHD group. The effect size of the difference between the EMHD patients and the HC individuals was large for the delta relative power (Hedge’s g = 0.91), alpha relative power (Hedge’s g = 1.00), and beta2 relative power (Hedge’s g = 1.16).

**Figure 3:**
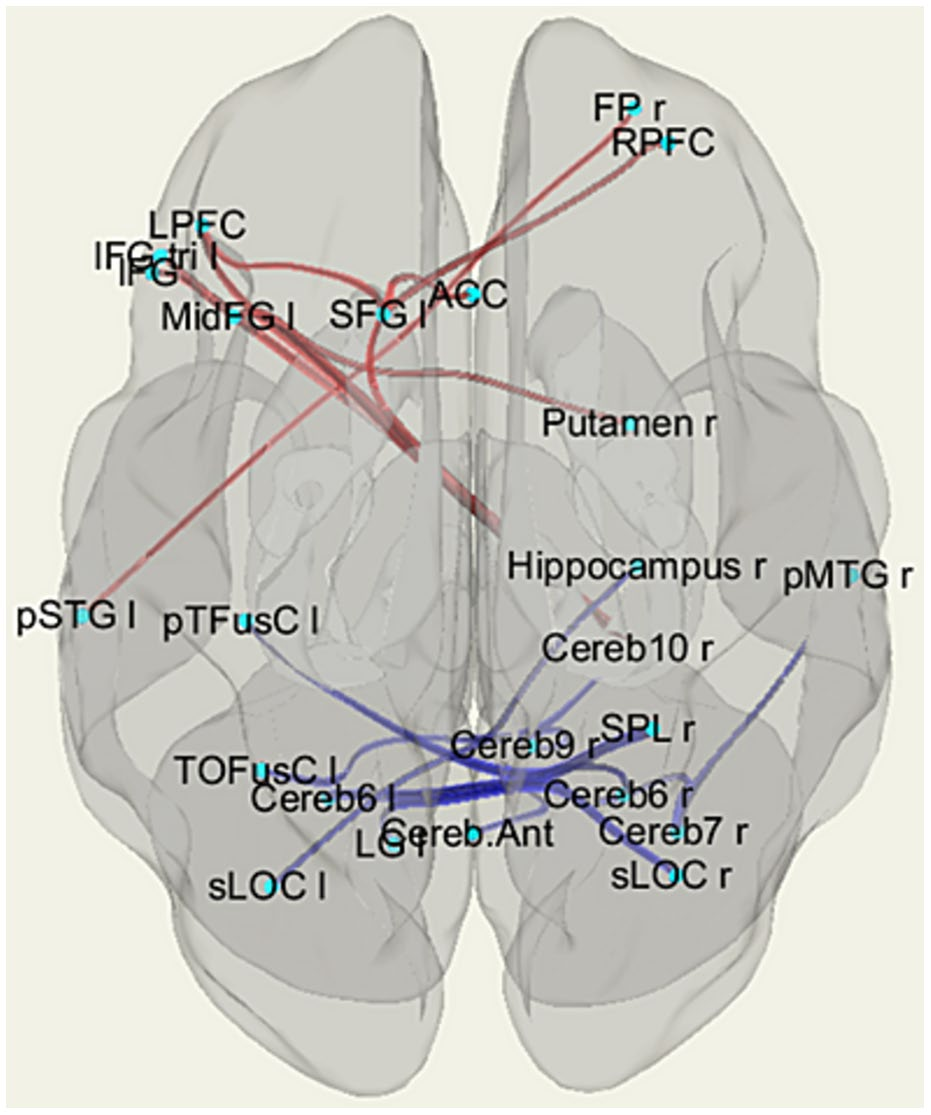
The patterns of functional links corresponding to the differences in fMRI resting-state functional connectivity (rsFC) among the individuals with HTT mutations, including those with PreHD and EMHD, and the control group of healthy individuals (p-FDR < 0.05). Abbreviations are the same as in Table 3.

ANOVA yielded significant effect of group on the relative power (preHD vs. healthy controls) of the 1 Hz spectral frequency bands in the range 4–13 Hz (*F*[2.91] = 18.32, *p* < 0.000001). A significant interaction effect was observed between the group and band factors (*F*[16.728] = 2.79, *p* = 0.0002). *Post hoc* comparisons showed that in the PreHD compared to the healthy controls, the relative power was significantly reduced in the 7–8 Hz (*p* = 0.03) and in the 8–9 Hz (*p* = 0.02) frequency bands (Figure 3). The effect size of the difference between the PreHD individuals and the HC individuals was significant in the 7–8 Hz (Hedge’s *g* = 0.76) and 8–9 Hz (Hedge’s g = 0.63) frequency ranges.

The patients with EMHD, in comparison to healthy controls, exhibited a significant reduction in the relative power within the 7–8 Hz (*p* = 0.01), 8–9 Hz (*p* = 0.0006), and 9–10 Hz (*p* = 0.0003), 10–11 Hz (*p* = 0.0002) (Figure 2B). The patients with EMHD, in comparison to PreHD individuals, showed a significant reduction in the relative power within the 8–9 Hz (*p* = 0.04), 9–10 Hz (*p* = 0.01), and 10–11 Hz (*p* = 0.003) ranges (Figure 2B). The effect size of the difference between the EMHD patients and the HC individuals was substantial in the 7–8 Hz (Hedge’s *g* = 1.22), 8–9 Hz (Hedge’s *g* = 1.67), 9–10 Hz (Hedge’s *g* = 1.105), and the 10–11 Hz (Hedge’s *g* = 0.85) frequency ranges.

An analysis of the correlation between the number of CAG repeats in the *HTT* gene and EEG parameters revealed a positive correlation between the number of CAG repeats and the delta and theta relative power (*r* = 0.46, *p* = 0.001; *p-FDR* = 0.005; *r* = 0.41, *p* = 0.002, *p-FDR* = 0.008 for delta and theta, respectively). The alpha relative power was inversely correlated with the number of CAG repeats (*r* = −0.43, *p* = 0.001, *p-FDR* = 0.005). The value for the correlation of DBS with the alpha relative power was *r* = −0.28, *p* = 0.04, *p*-FDR > 0.05.

The number of CAG repeats was inversely correlated with the relative power of the alpha subband at 8–9 Hz (*r* = −0.36, *p* = 0.009, *p-FDR* = 0.021) The relative power of the 7–8 Hz subband was not associated with the number of CAG repeats, but the difference in relative power of the 7–8 Hz and 4–5 Hz frequency subbands (7–8 Hz% minus 4–5 Hz%) showed significant negative correlation with the number of CAG repeats (*r* = −0.54, *p* = 0.00004, *p-FDR* = 0.0006). A significant negative correlation was also revealed between the DBS and the relative power in 8–9 Hz range (*r* = −0.38, *p* = 0.005, *p-FDR* = 0.013) as well as the difference in the relative power of the 7–8 Hz and 4–5 Hz frequency subbands (7–8 Hz% minus 4–5 Hz%) (*r* = −0.41, *p* = 0.003, *p*-*FDR* = 0.01).

We found significant correlation between the number of CAG repeats in *HTT* and age: *r* = −0.34, *p* = 0.012. To control for the potential influence of age on the correlations between CAG and EEG, we performed partial correlation analysis. The results have shown a significant partial correlation between CAG repeats and the relative power of the alpha and delta bands (*r* = −0.4, *p* = 0.003 and *r* = 0.41, *p* = 0.002 for alpha and delta respectively), as well as with the power of 1-Hz range 8–9 Hz (*r* = −0.42, *p* = 0.001), and the difference in relative power between the 7–8 Hz and 4–5 Hz subbands (*r* = −0.54, *p* = 0.0001.

In *HTT* mutation carriers, word fluency (FAS) scores showed inverse correlation with the delta (*r* = −0.35, *p* = 0.01) relative power and positive correlation with the alpha relative power (*r* = 0.37, *p* = 0.007). Analysis of correlation between spectral power of 1-Hz EEG frequency bands and psychometric characteristics revealed a tendency toward positive correlation between verbal fluency and relative power of the alpha subband at 8–9 Hz (*r* = 0.31, *p* = 0.025), and a tendency toward negative correlation between verbal fluency and relative power of the theta subband 4–5 Hz (*r* = −0.30, *p* = 0.03). The relative power of the 7–8 Hz subband correlated with total correct on memory working test (*r* = 0.44, *p* = 0.008). However, none of the correlations between EEG characteristics and psychometric scores survived FDR correction for multiple comparisons. No significant correlation was observed between relative power of any of the examined frequency bands and neuropsychological characteristics in the healthy control group.

### Comparison of fMRI resting-state functional connectivity among a group of individuals carrying an HTT mutation, including those with PreHD and EMHD, and a control group of healthy individuals

In addition to all other examinations, fMRI rsFC was assessed in a subgroup of 29 *HTT* mutation carriers with a CAG repeat length of 44.1 ± 0.7 and 25 age-matched healthy subjects. Their demographic and psychometric characteristics of the participants who underwent fMRI evaluation were similar to those from the main groups (Table 2).

**Table 2:** Demographic and psychometric characteristics of participants with fMRI evaluation.

	*HTT* mutation carriers							Healthy Control
	PreHD	*p* PreHD vs. HC	EMHD	*p* EMHD vs. PreHD	*p* EMHD vs. HC	Combined PreHD & EMHD	*p* HC vs. PreHD& EMHD	
N	19		10			29		25
Age, years (SE)	34.8 (1.4)	0.11	36.0 (3.0)	0.68	0.42	35.2 (1.4)	0.11	38.9 (1.9)
Male/female	7/12	0.74	7/3 ^a^	0.09	0.04	14/15	0.22	8/17
Education, years (SE)	14.7 (0.3	0.31	14.3 (0.4)	0.39	0.07	14.6 (0.2)	0.12	15.1 (0.2)
CAG repeats (SE)	43.0 (0.6)		46.3 ^b^ (1.5)	0.02		44.1 (0.7)		-
DBS (SE)	259.0 (19.4)		357.4^b^ (31.6)	0.009		292.9 (19.6)		-
FAS, words (SE)	51.0 (2.7)	0.47	31.9 ^a^^,^^b^ (2.3)	0.0001	<0.0001	44.6 ^a^ (2.6)	0.03	53.9 (3.0)
LMWT (SE)	5.5 (0.4)	0.82	4.6 (0.4)	0.14	0.04	5.2 (0.3)	0.34	5.6 (0.3)
LMWT total score (SE)	37.8 ^a^ (1.8)	0.01	33.3 ^a^ (2.0)	0.12	<0.0001	36.2 ^a^ (1.4)	<0.001	43.1 (0.8)
Pictures total score (SE)	35.3 ^a^ (0.8)	0.01	28.3 ^a^^,^^b^ (2.2)	0.001	<0.0001	33.4 ^a^ (1.0)	0.001	38.6 (0.8)
MMSE (SE)	29.5 (0.3)	0.07	26.2 ^a^^,^^b^ (1.1)	0.002	0.0001	28.3 ^a^ (0.5)	0.02	29.9 (0.1)
STAI state score (SE)	38.9 (2.4)	0.36	33.9 (3.9)	0.26	0.65	37.1 (2.1)	0.67	35.8 (2.2)
STAI trait score (SE)	41.9 (2.8)	0.63	36.5 (4.4)	0.29	0.36	40.1 (2.4)	0.95	40.3 (1.8)
UHDRS-TMS (SE)	1.3 (0.5)	0.011	18.6^a^^,^^b^ (2.7)	<0.001	<0.001	8.4 ^a^ (2.4)		0
SDMT (SE)	48.0 ^a^ (3.9)	0.003	29.6 ^a^^,^^b^ (3.2)	0.002	<0.0001	39.7 ^a^ (3.3)	<0.001	61.1 (1.9)
Stroop color naming (SE)	75.8 (4.8)	0.30	55.6 ^a^^,^^b^ (3.9)	0.004	<0.0001	66.5 ^a^ (3.7)	0.006	81.1 (2.3)
Stroop word reading (SE)	88.5 (3.4)	0.3	71.6 ^a^^,^^b^ (5.7)	0.014	0.001	81.2 ^a^ (3.5)	0.02	93.1 (2.8)
Stroop interference (SE)	42.8 (2.9)	0.56	35.0 ^a^ (2.5)	0.06	0.02	39.2 (2.1)	0.09	45.2 (2.8)
TFC (SE)	13.0 0.00		12.2 (0.3)	0.006	0.02	12.6 (0.2)	0.002	13.0 0.00

Data are presented as the means and standard errors (SE). MMSE, Mini-Mental State Examination; LMWT, Luria Memory Words Test; FAS, Controlled Oral Word Association Test; SST, STAI, Spielberger State–Trait Anxiety Inventory, SDMT, Symbol Digit Modalities Test; TFC – total functional capacity.

a significant differences *p* < 0.05 between healthy control and HTT mutation carriers.

b significant differences *p* < 0.05 between PreHD individuals and EMHD patients.

The ROI-to-ROI significant fMRI rsFC differences between the *HTT* mutation carriers and healthy controls are presented in Figure 3 and in Table 3. The positive rsFC in the following circuits of the frontal cortex was higher in HC individuals than in the *HTT* mutation carriers: the left superior frontal gyrus (SFG l) – the right rostral prefrontal cortex (RPFC r, salience network); the left superior frontal gyrus (SFG l) the left prefrontal cortex (LPFC l); the left superior frontal gyrus (SFG l) the anterior cingulate cortex (ACC, salience network) and the right frontal pole (FPr) –the left superior temporal gyrus (pSTG l). The positive rsFC was also higher in several fronto-cerebellar circuits in HC individuals than in the *HTT* mutation carriers.

**Table 3:** ROI-to-ROI resting state functional connectivity (rsFC) according to fMRI in individuals with HTT mutations, including those with PreHD and EMHD, and a control group of healthy individuals.

N	Analysis Unit	Groups	Beta	T	*p*-unc	*p*-FDR	Effect size Hedge g
1	MidFG l – Putamen r	*HC* vs. *HTT* *HC* *HTT*	0.16 0.06 −0.11	3.78 1.54 −4.36	0.00041 0.137 0.00016	0.034 0.23 0.0014	1.144
2	SFG l – LPFC l	*HC* vs. *HTT* *HC* *HTT*	0.19 0.59 0.4	3.47 14.38 10.91	0.001 <0.0000 <0.0000	0.043 <0.0000 <0.0000	0.985
**3**	SFG l –Salience RPFC r	*HC* vs. *HTT* *HC* *HTT*	0.18 0.19 0.01	4.12 5.47 0.27	0.00014 0.000013 0.79	0.022 0.00013 0.91	1.106
4	SFG l – Salience ACC	*HC* vs. *HTT* *HC* *HTT*	0.22 0.31 0.09	3,73 8.93 1.93	0.0005 <0.0000 0.064	0.026 <0.0000 0.18	1.048
5	FP r–pSTG l	*HC* vs. *HTT* *HC* *HTT*	0.19 0.2 0.01	4.03 6.03 0.29	0.0002 0.000003 0.77	0.03 0.00002 0.85	1.253
6	Cereb10 r – SFG l	*HC* vs. *HTT* *HC* *HTT*	0.17 0.18 0.00	3.74 5.05 0.16	0.00046 0.00004 0.87	0.026 0.0003 0.95	1.042
7	IFG tri l – Cereb10 r	*HC* vs. *HTT* *HC* *HTT*	0.23 0.18 −0.05	4.92 4.58 −1.78	<0.00001 0.00012 0.09	0.0015 0.00055 0.2	1.415
8	LPFC l – Cereb10 r	*HC* vs. *HTT* *HC* *HTT*	0.19 0.23 0.04	4.40 7.93 1.25	0.0005 <0.00001 0.22	0.0009 <0.00001 0.36	1.23
9	MidFG l – Cereb10r	*HC* vs. *HTT* *HC* *HTT*	0.2 0.22 0.03	4.35 6.84 0.94	0.000064 <0.00001 0.35	0.011 <0.00001 0.54	1.21
10	Cereb10 – Language IFG l	*HC* vs. *HTT* *HC* *HTT*	0.2 0.11 −0.1	4.26 2.71 −3.4	0.00009 0.012 0.002	0.014 0.034 0.009	1.20
11	Hippocampus r – sLOC l	*HC* vs. *HTT* *HC* *HTT*	−0.17 −0.1 0.07	−4.06 −3.39 2.38	0.00017 0.002 0.02	0.027 0.01 0.07	0.882
12	SPL r – pTFusC l	*HC* vs. *HTT* *HC* *HTT*	−0.21 −0.14 0.08	−3.92 −3.46 2.04	0.00021 0.0021 0.051	0.043 0.011 0.15	1.173
13	sLOC r – LG l	*HC* vs. *HTT* *HC* *HTT*	−0.21 −0.12 0.09	−3.69 −2.91 2.32	0.00054 0.0077 0.028	0.044 0.039 0.105	0.86
14	SPL r – Cereb6 r	*HC* vs. *HTT* *HC* *HTT*	−0.21 −0.11 0.1	−3.9 −2.52 3.03	0.0003 0.019 0.005	0.015 0.06 0.032	1.07
15	SPL r – Cereb6 l	*HC* vs. *HTT* *N* *HTT*	−0.23 −0.13 0.11	−3.99 −3.03 2.62	0.0002 0.0058 0.014	0.015 0.028 0.06	1.06
16	sLOC r – Cereb6 l	*HC* vs. *HTT* *N* *HTT*	−0.22 −0.17 0.05	−3.71 −3.73 1.21	0.00051 0.001 0.24	0.044 0.009 0.36	1.029
17	Cereb7 r – pMTG r	*HC* vs. *HTT* *HC* *HTT*	−0.2 −0.07 0.13	−3.79 −2.09 3.3	0.0004 0.05 0.0027	0.04 0.14 0.017	1.071
18	Cereb7 r – Cereb6 r	*HC* vs. *HTT* *HC* *HTT*	−0.22 −0.05 0.17	−3.72 −1.11 4.27	0.0005 0.28 0.0002	0.04 0.44 0.002	0.917
19	Cereb9 r –Cereb Ant Network	*HC* vs. *HTT* *HC* *HTT*	−0.22 0.16 0.37	−3.88 3.31 11.67	0.0003 0.003 <0.00001	0.049 0.019 <0.00001	1.068

MidFG l, Middle Frontal Gyrus left; SFG l, Superior Frontal Gyrus left; LPFC l, Lateral Prefrontal Cortex left; RPFC r, Rostral Prefrontal Cortex right; ACC, Anterior; Cingulate Cortex; FP r, Frontal Pole right; pSTG l, Posterior Superior Temporal Gyrus left; Cereb10 r, Cerebellum 10 right; IFG tri l, Inferior Frontal Gyrus Triangularis left; sLOC l, Superior Lateral Occipital Cortex left; SPL r, Superior Parietal Lobule right; pMTG r, Posterior Middle Temporal Gyrus right; Cereb6 l, Cerebellum 6 left; Cereb 9 r, Cerebellum9 right; Cereb Ant Network, Cerebellar Anterior Network.

HC, resting state functional connectivity (rsFC) characteristics of the healthy control individuals; HTT, rsFC characteristics of the HTT mutation carriers; HC vs. HTT, difference between rsFC characteristics in the healthy controls and in the HTT mutation carriers.

Beta, regression coefficient.

The healthy controls had stronger negative rsFC than the *HTT* mutation carriers in the following interhemispheric circuits: the right hippocampus (Hippocampus r)– the left superior lateral occipital cortex (sLOC l); the right superior parietal lobule – the left posterior temporal fusiform cortex (pTFusC l); and the right superior lateral occipital cortex (sLOC r) – the left lingual gyrus (LG l). The negative rsFC was also higher in HC individuals than in the *HTT* mutation carriers in several cortico-cerebellar circuits, between the cerebellum and the following posterior cortical regions: sLOC r, SPL r, right posterior middle temporal gyrus (pMTG r).

The *HTT* mutation carriers also had stronger negative rsFC than the healthy controls between the right putamen and the contralateral frontal cortex (MidFG l).

The effect size of the difference in fMRI rsFC between the *HTT* mutation carriers and the HC was substantial, exceeding 0.8 (Table 3).

The PreHC individuals had higher positive rsFC than the patients with EMHD in the following circuits: the left rostral prefrontal cortex (RPFC l, salience network) – the left posterior superior temporal gyrus (pSTG l, language network (beta = 0.26, T = 3.63, *p-*FDR = 0.048); the left middle temporal gyrus (pMTG l) and the left rostral prefrontal cortex (RPFC l) (beta = 0.29, T = 3.8, *p*-FDR = 0.034).

The positive rsFC between the left postcentral gyrus (PostCG l) and left sensorimotor lateral cortex was lower in the PreHD individuals than in the patients with EMHD ((beta = −0.38, T = −4.67, *p*-FDR = 0.012).

The PreHC individuals had stronger negative rsFC than the patients with EMHD in the following circuits: the left lingual gyrus (LG l) and the left posterior superior temporal gyrus (pSTG l) (beta = −0.24, T = -4.31, *p*-FDR = 0.03). Negative rsFC was also higher in PreHD individuals than in *EMHD* patients in several cortico-cerebellar circuits: the left cerebellum 45 (cereb45 l) – the left posterior middle temporal gyrus (pMTG l) (beta = −0.31, T = -5.23, *p*-FDR = 0.0027); the vermis45 – the left posterior temporal gyrus (pSTG l) (beta = −0.28, T = -4.87, *p*-FDR = 0.007); the vermis45 – left angular gyrus (AG l)) (beta = −0.26, T = -3.87, *p*-FDR = 0.034); the vermis45 – left supramarginal gyrus (SMG l)) (beta = −0.24, T = -3.78, *p*-FDR = 0.034); the vermis45 – left middle temporal gyrus (pMTG l)) (beta = −0.24, T = -3.68, *p*-FDR = 0.034).

### Association of EEG spectral power with fMRI functional connectivity of brain networks in HTT mutation carriers and healthy controls

Regression analysis between EEG alpha relative power and fMRI was conducted on *HTT* mutation carriers and HC subjects who underwent both examinations. The subgroup included 28 *HTT* mutation carrier (14 men, 14 women), a mean age 35.3 ± 1.4 years with a CAG repeat length of 44.2 ± 0.7 and 17 HC subjects (4 men, 13 women), a mean age 39.9 ± 2.2 years. The group *HTT* mutations carriers included 18 PreHD individuals (7 men, 11 women), a mean age 34.9 ± 1.5 years with CAG repeat length of 43.0 ± 0.6; and 10 individuals with EMHD (7 men and 3 women), a mean age of 36.0 ± 3.0 years with a CAG repeat length of 46.3 ± 1.5.

**Figure 4:**
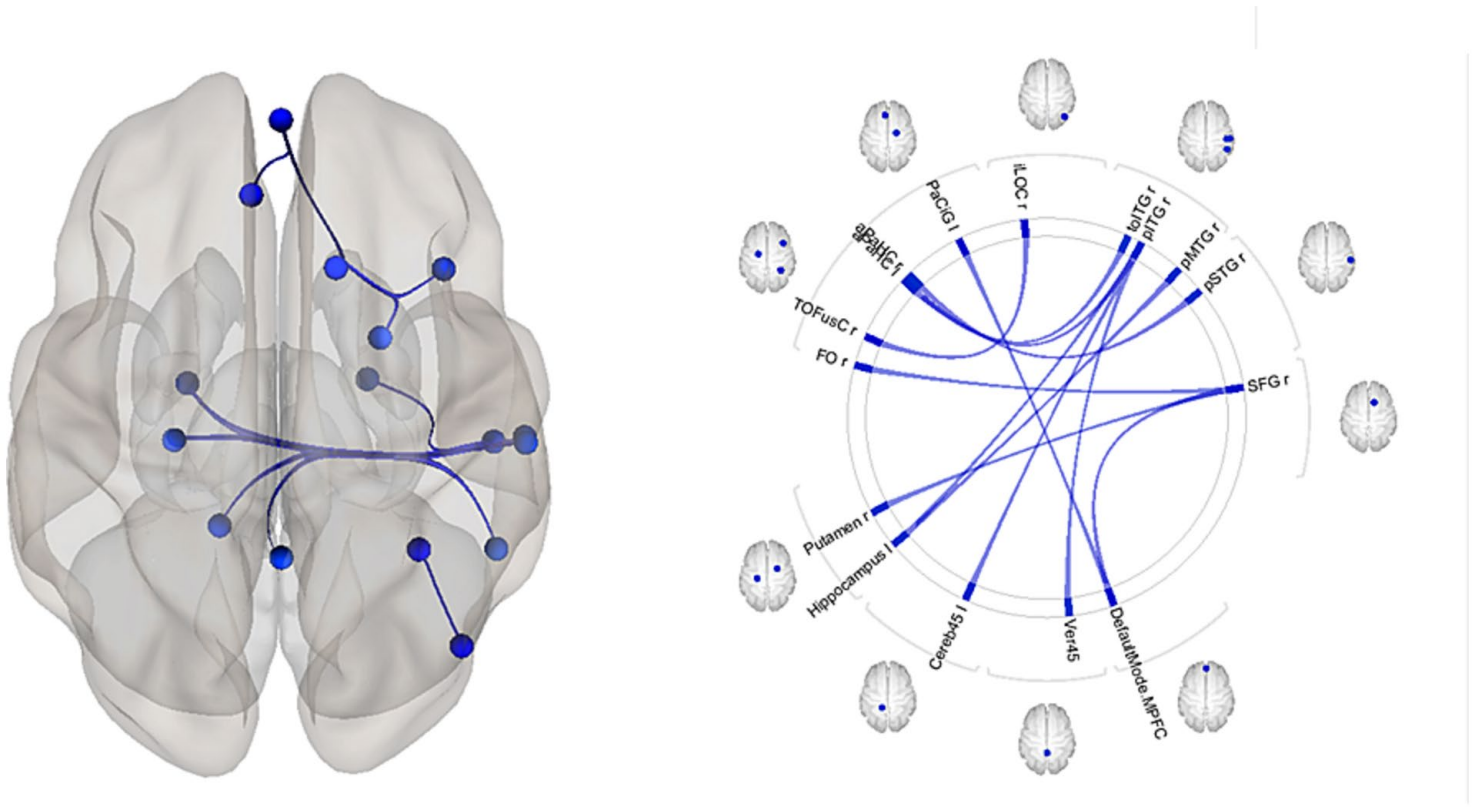
Patterns of circuits in which fMRI resting-state functional connectivity (rsFC) is associated with EEG alpha relative power among the Huntington’s disease (HD) mutation carriers and healthy individuals. Results of regression analysis. Abbreviations are the same as in Table 4.

In the overall sample that included both *HTT* mutation carriers and healthy control participants, reduced alpha relative power was associated with increased connectivity in several brain circuits (Table 4, Figure 4). The rsFCs of the frontal cortex included the MPFC.DMN-related networks and the putamen-frontal network. The occipital cortex circuits comprised rsFC between the right inferior lateral occipital cortex (iLOC r) and the right temporo-occipital fusiform cortex (TOFusC r). The networks of the temporal cortex included the rsFC between the right superior temporal cortex (pSTG r) and the right parahippocampal cortex (PaHC), as well as the interhemispheric connections involving the left hippocampus with the right posterior middle temporal gyrus (pMTG) and the right posterior inferior temporal gyrus (pITG). Additionally, they involved connectivity between the right pITG and the PaHC l and cerebellum.

**Table 4:** Regression analysis results showing the association between EEG alpha relative power and fMRI resting-state functional connectivity.

Analysis Unit	*T*	*p-unc*	*p-FDR*
SFG r – DMN MPFC	−4.23	0.0001	0.0197
SFG r – FO r	−3.69	0.0006	0.0389
DMN MPFC– PaCiG l	−3.92	0.0003	0.0254
pMTG r – Hippocampus l	−4.07	0.0002	0.0319
pSTG r – aPaHC r	−4.51	0.0000	0.0080
pITG r – aPaHC l	−4.31	0.0001	0.0152
pITG r– Hippocampus l	−3.81	0.0004	0.0235
aPaHC l – toITG r	−3.89	0.0003	0.0277
pITG r – Cereb45 l	−3.85	0.0004	0.0235
pITG r – Ver45	−3.66	0.0007	0.0279
TOFusC r – iLOC r	−4.06	0.0002	0.0331
SFG r – Putamen r	−3.64	0.0007	0.0389

r, right; l – left; DMN MPFC, DefaultMode Medial Prefrontal Cortex; FO, Frontal Operculum; pITG, Inferior Temporal Gyrus, posterior division; iLOC, Lateral Occipital Cortex inferior division; pMTG, Middle Temporal Gyrus, posterior division; PACiG, Paracingulate Gyrus; aPaHC, Parahippocampal cortex, anterior division; pPaHC, parahippocampal cortex posterior division; SFG, Superior Frontal Gyrus; pSTG, Superior Temporal Gyrus, posterior division; TOFusC, Temporal Occipital Fusiform Cortex; pITG, Inferior Temporal Gyrus, posterior division; toITG, Inferior Temporal Gyrus, temperooccipital division; Cereb, Cerebellum, Ver, Vermis.

## Discussion

The main results of the present study revealed significant alterations in EEG activity, particularly in the alpha band and narrow-band activity within the 7–9 Hz range, in individuals with PreHD and EMHD when compared to the healthy control group. The study found significant alterations in fMRI rsFC in cortico-cortical, putamen-cortical, and cortico-cerebellar neural networks among individuals who were *HTT* mutation carriers, in comparison to the HC group. Further analysis has enabled us to identify brain networks in which fMRI functional connectivity was associated with EEG alpha power in the *HTT* mutation carriers and HC individuals.

### EEG alterations in PreHD and EMHD

We found that in the group of *HTT* mutation carriers, including individuals with PreHD and EMHD, there was a significant decrease in the relative power of both the alpha band and narrow-band activity specifically within the range of 7–10 Hz, when compared to the HC group. In the comparison between individuals with PreHD and HC, the only significant alteration observed was a decrease in the relative power of EEG within a narrow frequency range at the theta-alpha border and slow alpha activity (7–9 Hz), with Hedge’s g effect sizes of 0.76 and 0.63 for the 7–8 Hz and 8–9 Hz, respectively. These effect sizes, while not extremely large, still indicate a notable and statistically meaningful distinction between the two groups within these specific frequency bands. These results suggest that these frequency ranges may be particularly relevant for understanding the differences in neural activity between individuals with PreHD and those without the condition.

In the EMHD patients, apart from the more pronounced decrease in power within the7-10 Hz, a significant reduction in power within the classic alpha band was also detected in the present study. The difference in alpha relative power between EMHD and HC individuals was substantial, with an effect size of 1.0. Previous studies have consistently reported a reduction in alpha power in individuals with HD (de Tommaso et al., 2003; Bellotti et al., 2004; van der Hiele et al., 2007; Hunter et al., 2010; Painold et al., 2010, 2011; Leuchter et al., 2017; Piano et al., 2017; Odish et al., 2018; Delussi et al., 2020). The reduction in alpha power observed in the EMHD group may reflect a dysregulation of cortical excitability due to the alteration of inhibitory GABAergic mechanisms and may also be associated with excitotoxicity (Palva and Palva, 2007).

Previous studies have reported a decrease in power at the theta-alpha border in individuals with preHD and EMHD (Painold et al., 2011; Ponomareva et al., 2014; Hawellek et al., 2022).

This frequency range, 7–9 Hz, has been associated with thalamo-cortical mechanisms involved in cognitive functions. A decrease in 7–8 Hz activity has been observed during the retrieval of semantic memories (Slotnick et al., 2002), whereas this activity typically increases during a state of deep relaxation and reverie (Sokhadze et al., 2008). These results suggest a potential link between the decline in 7–8 Hz power and cortical disinhibition in preHD and HD patients.

Furthermore, a decrease in power within this frequency range has also been observed during sleep in preHD and EMHD patients (Lazar et al., 2015). Researchers have suggested that this decrease may underlie deficits in neural restoration following wakefulness. The hypothesis linking 7–8 Hz activity to the processes of HD development was supported by evidence that the use of the huntingtin-lowering oligonucleotide tominersen led to a partial recovery of activity within this frequency range (Hawellek et al., 2022).

In the EMHD group, we found an increase in beta2 activity. Previous EEG studies in HD, including the research by Bylsma et al. (1994) identified an increase in beta activity, while contrasting results were found in other studies (Painold et al., 2011; Piano et al., 2017). The discrepancies in the alteration of the beta band may be related to the heterogeneity of HD among patients and variations in the EEG analysis methods used. In our study, as well as in the research of Bylsma et al. (1994), normalization was applied to the analysis of EEG spectral power. Normalization minimizes interindividual variability in absolute power, which arises from factors such as skull thickness and impedance, and allows for a better comparison of an individual’s power distribution across frequency bands. However, a reduction in the spectral power of the alpha band found in EMHD can result in an increase in the relative spectral power of other frequency ranges, especially in the beta bands, which typically have lower power. Results obtained from the R6/2 mouse model of HD show an increase in beta activity during the disease progression (Fisher et al., 2013, 2016). Further research is needed to clarify whether an increase in beta activity may indeed occur in some patients during specific stages of HD progression.

We observed a correlation between an increase in delta power and a decrease in alpha power, and poorer performance on cognitive tests, particularly verbal fluency. We also revealed a positive correlation between the relative power of the 7–8 Hz sub-band relative power and performance on memory working test. The correlations between EEG characteristics and psychometric scores did not survive FDR correction for multiple comparisons and should be considered as tendencies.

These findings align with prior studies that have shown a connection between a reduction in alpha and increase in delta power and cognitive decline in individuals with HD (Bylsma et al., 1994; Painold et al., 2010, 2011; Ponomareva et al., 2014; Odish et al., 2018). Changes in spectral power in individuals with HD have been found to correlate with performance on cognitive tests, including the Mini Mental Status Examination, FAS verbal fluency test, Stroop Word Reading Test, and the Symbol Digit Modalities Test.

In the present study a positive correlation was found between the length of the CAG repeats in the *HTT* gene and the relative power of delta and theta frequencies in *HTT* mutation carriers. On the other hand, the CAG repeat length was negatively correlated with the alpha band and its subbands. Additionally, a negative correlation was found between the difference in relative power of the 7–8 Hz and 4–5 Hz and the number of CAG repeats. These results validate our prior findings on a larger sample size (Ponomareva et al., 2014). Previous research has also revealed a correlation between EEG characteristics, such as the relative delta anterior–posterior gradient, and the number of CAG repeats (Nguyen et al., 2010, for review, Hunter et al., 2010; Piano et al., 2017, for review).

### Alterations in fMRI resting-state functional connectivity in HTT mutation carriers

Numerous studies of PreHD and EMHD have employed data-driven approaches to explore whole-brain connectivity and these studies have consistently demonstrated altered functional connectivity patterns (Dumas et al., 2013; Harrington et al., 2015; Poudel et al., 2015; Sánchez-Castañeda et al., 2017; Espinoza et al., 2018; Pini et al., 2020; Polosecki et al., 2020). The results from these studies have shown a range of differences, which can be attributed to the variation in the cohorts studied and the methodologies used in the investigations.

Evidence from fMRI studies indicates the presence of distributed anticorrelated networks in the brain (Fox et al., 2005; Martinez-Gutierrez et al., 2022). Task-positive networks, such as the frontoparietal and salience networks, are involved in cognitive control and attention modulation, becoming more active during external goal-directed tasks. On the other hand, the DMN, crucial for memory function, shows reduced activity during external goal-directed tasks (Sheline and Raichle, 2013). In healthy adults, rsFC between task-positive and task-negative networks is typically characterized by negative correlation.

The current study identified significant changes within cortico-cortical, putamen-cortical, cortico-hippocampal, and cortico-cerebellar networks in individuals carrying *HTT* mutations, when compared to healthy controls. Altered connectivity was seen in networks that show both negative and positive connectivity in healthy individuals. The effect size for the difference in fMRI rsFC in these circuits between *HTT* mutation carriers and the HC was notably large, exceeding 0.8.

HD is primarily characterized by the progressive degeneration of the striatum, with the putamen being one of the key regions affected. This neurodegeneration is largely attributed to the loss of GABAergic medium spiny neurons (Spampanato et al., 2008). We found a significant difference in rsFC between the right putamen and the left middle frontal gyrus when comparing *HTT* mutation carriers to healthy controls. In *HTT* mutation carriers this circuit displayed negative rsFC, while connectivity values in HC individuals were not significantly different from zero. Previous studies demonstrated reduced rsFC in fronto-striatal circuits in preHD and HD patients (Poudel et al., 2015; Espinoza et al., 2018; Nair et al., 2022). However, Kronenburger et al. (2019) found increased striatum-prefrontal connectivity in premanifest HD subjects. The authors suggested that enhanced rsFC may imply a compensatory mechanism, where additional cortical regions are recruited to subserve functions that have been impaired due to HD pathology.

Neurodegeneration in the frontal lobe is a prominent feature of HD (Aylward et al., 1998; Matsui et al., 2014). Prior studies have reported aberrant rsFC patterns in the frontal lobe of HD patients (Thiruvady et al., 2007; Harrington et al., 2015; Langley et al., 2021). Reduced rsFC was found within the bilateral superior frontal gyrus between ventral attentional center and the frontal area (Zhang et al., 2023, for review). However, other studies have found an increase in frontal resting-state functional connectivity (rsFC) and enhanced frontal-posterior rsFC (Werner et al., 2014; Harrington et al., 2015). The current study showed that in *HTT* mutation carriers, compared to healthy controls, a significant decrease in positive rsFC is observed in several cortical networks, particularly within the frontal lobes. Specifically, decreased rsFC was found between the left superior frontal lobe and the regions within salience networks.

We have revealed aberrant cortico-cerebellar rsFC in *HTT* mutation carriers compared to HC individuals. In accordance with prior research, the findings of the present study identified aberrant connectivity between the cerebellum and the cortical regions implicated in executive functions, including the prefrontal and parietal cortices (Wolf et al., 2015; Sarappa et al., 2017; Franklin et al., 2021). These findings align with the disturbance of executive function in HD, supporting the notion of involvement of the cerebellum in the nonmotor symptoms of the disease.

In the current study, we found diminished negative rsFC in individuals with preHD compared to the healthy controls, particularly in interhemispheric networks. Specifically, we observed reduced rsFC between the right superior parietal lobule (SPL) and the left posterior fusiform cortex (pTFusC), between the right lateral occipital cortex (sLOCr) and the left lingual gyrus (LGl), and between the right hippocampus and the left sLOC. Previous studies have suggested the importance of abnormal interhemispheric connectivity in the pathophysiology of HD (Harrington et al., 2015).

### Association of EEG power and fMRI connectivity in HTT mutation carriers and healthy controls

Our findings indicate a prominent prevalence of neural networks showing an inverse correlation between fMRI connectivity patterns and EEG alpha relative power in *HTT* mutation carriers and HC individuals. This finding aligns with the results of previous studies that have reported a negative association between the functional connectivity of cortical circuits and alpha power (Scheeringa et al., 2012; Timmermann et al., 2023).

In particular, we found a significant relationship between alpha power and the rsFC of circuit located in frontal regions and the putamen. Studies show that the putamen is involved in modulating alpha rhythm (Hughes and Crunelli, 2005). Early dysfunction and degeneration of the putamen in HD may influence the alpha rhythm and may underlie the association of fMRI rsFC and alpha power. Our results are consistent with studies suggesting that the impact on the cortico-striato-thalamo-cortical circuitry is an important factor in the abnormal reduction of intrinsic alpha oscillations observed throughout the cortex in HD (Hughes and Crunelli, 2005; Alper et al., 2006). Furthermore, the degeneration of GABAergic projections from the putamen to the frontal cortex can impair inhibitory function and contribute to the dysregulation of cortical rhythms (Smith et al., 1998).

The present study’s results indicated that the rsFC between the MPFC, a central hub within the DMN, and the SFG *r* was associated with a decrease in alpha rhythm. Previous studies have reported that the MPFC and the SFGr are involved in the pathophysiology of HD (Wolf et al., 2012; Pini et al., 2020). Accumulating evidence suggests that the MPFC and the SFG r work together as part of a cognitive control network (Mathewson et al., 2014). The MPFC provides contextual information and top-down signals, while the SFG r exerts cognitive control to suppress resting alpha rhythms across multiple cortical areas.

We also found significant association between a decrease of EEG alpha power and an increase of rsFC between the right inferior lateral occipital cortex (iLOC) and the right temporo-occipital fusiform cortex (TOFusC). It was consistently shown that lower alpha power indicates higher cortical excitability and lower perceptual threshold (Foxe and Snyder, 2011). Our results suggest that pathological process in HD may disrupt normal communication between visual areas, and that the reduction in alpha power could indicate compensatory increases in connectivity in response to disease-related changes.

Additionally, we found an association of the alpha power with the interhemispheric circuits between the left hippocampus with the pMTG r and the pITG r, as well as between the right pITG, the left PaHC and the cerebellum. Prior research has shown that hippocampal dysfunction worsens in a linear manner during the development of HD. Furthermore, studies have suggested that abnormalities in the hippocampus could serve as a marker for ongoing cognitive decline in individuals with HD (Harris et al., 2019). Our results indicate that the alteration of hippocampal networks may be a factor contributing to the reduction in alpha power in HD.

The precise mechanisms underlying the alteration of the alpha rhythm in HD are still not fully understood. The findings of the current study indicate that alteration in several cortico-cortical networks, including the putamen-frontal, DMN-related and cortico-hippocampal circuits, can contribute to the reduction in alpha power in HD.

The limitations of the present study include the relatively small number of participants, who underwent fMRI examination, especially in the EMHD group. This suggests that the results on the association of EEG and rsFC in HD are preliminary. Nevertheless, the findings on the interdependence of the EEG and fMRI characteristics can contribute to an understanding of the underlying mechanisms behind alpha rhythm abnormalities in *HTT* mutation carriers. Another limitation is the nonsimultaneous registration of EEG and fMRI. However, as in the resting state of these characteristics are relatively stable, separate EEG and fMRI recordings across experimental sessions are sufficient (Scrivener, 2021).

In conclusion, the study identified significant differences in EEG power between individuals with PreHD and healthy controls, with a notable decrease in power within a specific frequency range at the theta-alpha border and slow alpha activity (7–9 Hz). In EMHD patients, a reduction in classical alpha band power was observed compared to HC, while a decrease in relative power within the 7–9 Hz frequency range effectively distinguishes preHD individuals from healthy controls.

Furthermore, fMRI analysis revealed alterations in functional connectivity within various brain networks, particularly within the frontal lobe, putamen-cortical, and cortico-cerebellar networks, in individuals carrying the *HTT* mutation compared to healthy controls.

The analysis of the relationship between EEG and fMRI rsFC revealed a correlation between a decrease in alpha power, observed in individuals with EMHD, and a reduction in inhibitory control in cortico-cortical networks. Such networks include putamen-cortical, DMN-related and cortico-hippocampal circuits. These alterations in functional connectivity may contribute to the observed EEG changes in HD.

Overall, the findings suggest that EEG and fMRI provide valuable information about pathophysiological mechanisms of HD progression during preclinical and early manifest stages of the disease. A decrease in inhibitory control within the putamen-cortical, DMN-related and cortico-hippocampal circuits, accompanied by a reduction in alpha and theta-alpha border oscillatory activity, could potentially contribute to cognitive decline in HD.

## Data availability statement

The original contributions presented in the study are included in the article/supplementary materials, further inquiries can be directed to the corresponding author.

## Ethics statement

The studies involving humans were approved by Local Ethics Committees of Research Center of Neurology, Moscow, Russia. The studies were conducted in accordance with the local legislation and institutional requirements. The participants provided their written informed consent to participate in this study.

## Author contributions

NP: Conceptualization, Data curation, Formal analysis, Funding acquisition, Investigation, Methodology, Validation, Visualization, Writing – original draft, Writing – review & editing. SK: Data curation, Investigation, Writing – review & editing. NA: Data curation, Investigation, Writing – review & editing. RK: Data curation, Investigation, Writing – review & editing, Methodology. MK: Data curation, Investigation, Writing – review & editing. EK: Data curation, Investigation, Writing – review & editing. DM: Data curation, Investigation, Writing – review & editing. GU: Data curation, Writing – review & editing. EK: Data curation, Writing – review & editing. AM: Writing – review & editing, Investigation, Methodology. VF: Writing – review & editing, Investigation, Methodology. ER: Writing – review & editing, Conceptualization, Data curation. SI: Conceptualization, Writing – review & editing, Methodology, Supervision.
