# Corrigendum: Neurophysiological hallmarks of Huntington's disease progression: an EEG and fMRI connectivity study

**DOI:** 10.3389/fnagi.2024.1487201

**Published:** 2024-09-19

**Authors:** Natalya V. Ponomareva, Sergey A. Klyushnikov, Natalia Abramycheva, Rodion N. Konovalov, Marina Krotenkova, Ekaterina Kolesnikova, Daria Malina, Gusel Urazgildeeva, Elena Kanavets, Andrey Mitrofanov, Vitaly Fokin, Evgeny Rogaev, Sergey N. Illarioshkin

**Affiliations:** ^1^Research Center of Neurology, Moscow, Russia; ^2^Center for Genetics and Life Science, Sirius University of Science and Technology, Sochi, Russia; ^3^Research Center of Mental Health, Moscow, Russia; ^4^Department of Psychiatry, Umass Chan Medical School, Shrewsbury, MA, United States

**Keywords:** EEG, functional MRI, Huntington's disease, preclinical stage, HTT gene, CAG repeats, cognitive functions

In the published article, there was an error in the caption for Figure 3 and Figure 4, where these captions were erroneously switched. The corrected captions for Figure 3 and Figure 4 appear below:

Figure 3. The patterns of functional links corresponding to the differences in fMRI resting-state functional connectivity (rsFC) among the individuals with HTT mutations, including those with PreHD and EMHD, and the control group of healthy individuals (p-FDR < 0.05). Abbreviations are the same as in Table 3.

Figure 4. Patterns of circuits in which fMRI resting-state functional connectivity (rsFC) is associated with EEG alpha relative power among the Huntington's disease (HD) mutation carriers and healthy individuals. Results of regression analysis. Abbreviations are the same as in Table 4.

The authors apologize for these errors and state that this does not change the scientific conclusions of the article in any way. The original article has been updated.

